# A Stable Silanol Triad in the Zeolite Catalyst SSZ‐70

**DOI:** 10.1002/anie.202001364

**Published:** 2020-04-23

**Authors:** Christian Schroeder, Christian Mück‐Lichtenfeld, Le Xu, Nicolás A. Grosso‐Giordano, Alexander Okrut, Cong‐Yan Chen, Stacey I. Zones, Alexander Katz, Michael Ryan Hansen, Hubert Koller

**Affiliations:** ^1^ Institut für Physikalische Chemie Westfälische Wilhelms-Universität Corrensstrasse 28/30 48149 Münster Germany; ^2^ Center of Soft Nanoscience Westfälische Wilhelms-Universität Busso-Peus-Strasse 10 48149 Münster Germany; ^3^ Organisch-Chemisches Institut Westfälische Wilhelms-Universität Corrensstrasse 40 48149 Münster Germany; ^4^ Department of Chemical and Biomolecular Engineering University of California Berkeley CA 94720 USA; ^5^ Chevron Energy Technology Company Richmond CA 94804 USA

**Keywords:** defects, heterogeneous catalysis, silanols, solid-state NMR spectroscopy, zeolites

## Abstract

Nests of three silanol groups are located on the internal pore surface of calcined zeolite SSZ‐70. 2D ^1^H double/triple‐quantum single‐quantum correlation NMR experiments enable a rigorous identification of these silanol triad nests. They reveal a close proximity to the structure directing agent (SDA), that is, N,N′‐diisobutyl imidazolium cations, in the as‐synthesized material, in which the defects are negatively charged (silanol dyad plus one charged SiO^−^ siloxy group) for charge balance. It is inferred that ring strain prevents the condensation of silanol groups upon calcination and removal of the SDA to avoid energetically unfavorable three‐rings. In contrast, tetrad nests, created by boron extraction from B‐SSZ‐70 at various other locations, are not stable and silanol condensation occurs. Infrared spectroscopic investigations of adsorbed pyridine indicate an enhanced acidity of the silanol triads, suggesting important implications in catalysis.

## Graphical abstract

**Silanol defects** determine adsorption and catalysis in zeolites by their hydrophilic properties, but they can also contribute to catalytic reactions through their weak acidities or cooperative effects with other active sites. Well‐defined silanol defect clusters (see structure: cyan H, red O, black Si) with distinct size in topologically related zeolites, SSZ‐70 and ITQ‐1 are identified by NMR spectroscopy.

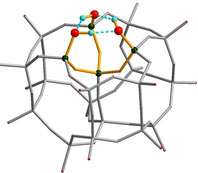

Zeolites are microporous materials comprising a 3D TO_4/2_ network of tetrahedral framework atoms (T=Si, Al, Ti, B, …). They are essential in numerous commercial applications, such as acid catalysis and redox chemistry.^1^ One of the main reasons for their high catalytic selectivity is that zeolites offer unique reaction spaces in their micropores, which control the reaction mechanism by adsorption effects. Silanol defects contribute to the hydrophilic properties and thus influence adsorption in these zeolite pores,^2^ but they can also be an integral part of the reaction center, such as for the Beckmann rearrangement of cyclohexanone oxime to ϵ‐caprolactame in ZSM‐5 or Beta zeolites.^3^ More recently, silanol groups were reported to contribute to oxidation catalysis, through cooperativity with Lewis acid sites.^4^ These effects include olefin epoxidation reactions in titanosilicates synthesized from SSZ‐70 precursors.^5^

There has been a longstanding debate in the literature regarding the nature of defect sites and their formation in zeolites.^6^ The removal of a T atom was suggested to create a so‐called silanol nest. However, this extraction typically requires acid treatment or steaming at elevated temperatures, resulting in condensation and hydrolysis reactions (migration of vacancies).^7^ Thus, such vacancies assemble in larger entities, either forming internal mesopores or disappearing on the crystal surface. Therefore, the stability of the postulated SiOH tetrad nests was subject to critical scrutiny.6e, 8 Clearly, so far, investigations lacked the methodology to rigorously determine the number of silanol groups in a defect site.

Herein we utilize double (or higher multi) quantum ^1^H MAS NMR experiments (MAS=magic angle spinning) to measure homonuclear dipolar interactions.^9^ We have previously applied such methods to investigate defect sites in as‐made zeolites,^10^ as well as Brønsted acid sites in zeolite Y.^11^ The ^1^H dipolar interactions directly probe the spatial proximities of silanol groups, enabling their cluster sizes to be determined within a distance range of typically 5 Å (to a maximum of 8 Å).

Zeolite SSZ‐70^12^ (framework type code: ^*^‐SVY)^13^ is related to zeolite ITQ‐1^14^ (MWW), which both consist of the same type of layers, Figure 1. SSZ‐70 is a partially disordered material due to disorder in the layer stacking. While these layers are fully interconnected in ITQ‐1 (Figure 1 b), they are shifted in SSZ‐70, resulting in structural Q^3^ groups with dangling silanols (Figure 1 a,c). Additionally, the atomic positions of Si and O atoms in these Q^3^ groups were refined with only 50 % occupancy in the Rietfeld analysis. Therefore, SiOH triad nests (Figure 1 c) were proposed, while hydrogen atoms could not be located.^12^

**Figure 1:**
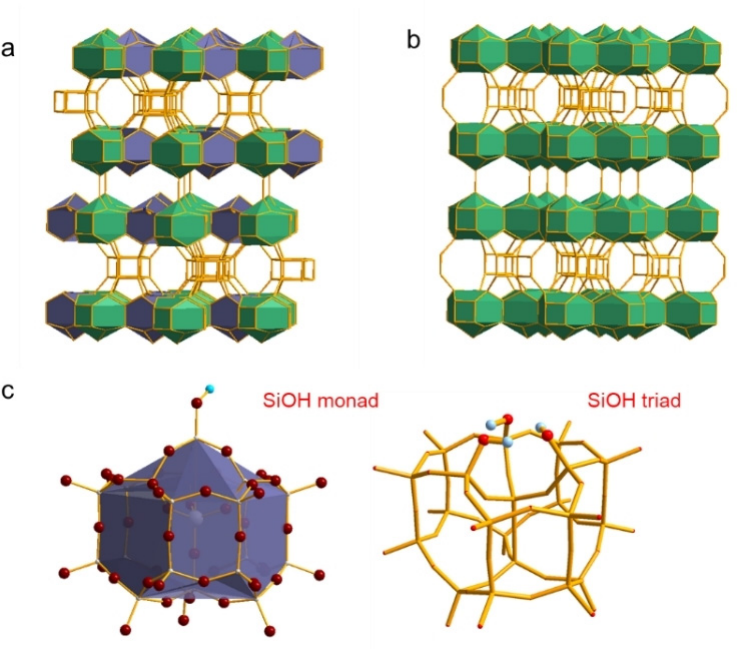
a) Framework topology of zeolite SSZ‐70, highlighting in different colors the cages, which are interconnected (green) or having silanol groups (blue), b) framework topology of ITQ‐1 with all cages interconnected, c) silanol monad and triad model; cyan H, red O. O atoms are omitted in models in (a) and (b).

The ^29^Si MAS NMR spectra of SSZ‐70 (Figure S1 a in the Supporting Information and Refs. 12, 15) and ITQ‐1^14^ are almost identical, except for the presence of Q^3^ defect sites in SSZ‐70 that do not exist in ITQ‐1. This similarity manifests that the layers are topologically identical in both zeolites. However, the two materials exhibit distinct defect site properties, as unveiled by the ^1^H double‐quantum single‐quantum (DQ‐SQ) correlation NMR spectra of SSZ‐70 (Figure 2 a) and ITQ‐1 (Figure 2 b), displaying clear differences in the silanol pairing pattern. These 2D experiments show in the SQ dimension the chemical shifts of the two protons that are in spatial proximity and the sum of their two chemical shifts is shown on the DQ axis.

**Figure 2:**
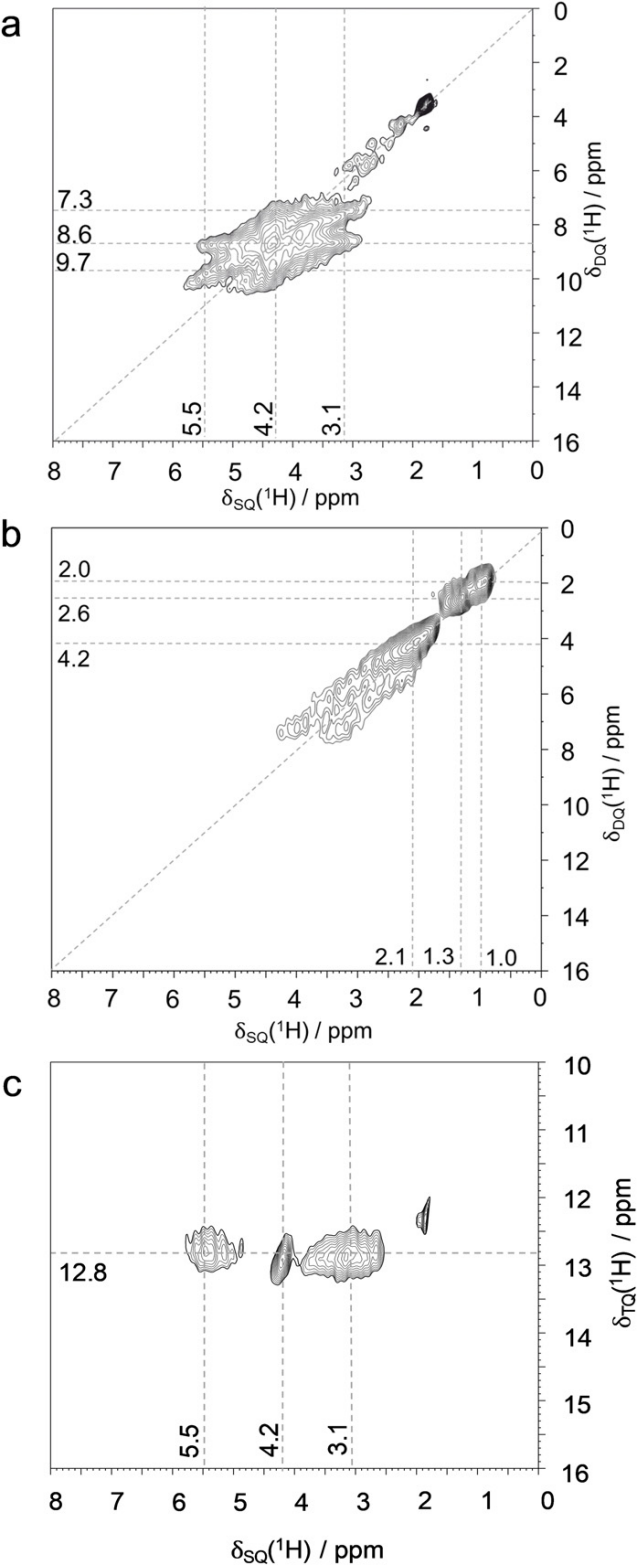
2D ^1^H DQ‐SQ MAS NMR spectra of calcined a) all‐silica zeolite SSZ‐70, b) ITQ‐1, c) 2D ^1^H TQ‐SQ MAS NMR spectrum of all‐silica SSZ‐70.

Analysis of 1D slices (Figure S3) taken from the 2D data of calcined SSZ‐70 (Figure 2 a) shows three major cross‐correlation signals at DQ chemical shifts of 7.3 ppm (3.1+4.2), 8.6 ppm (3.1+5.5), and 9.7 ppm (4.2+5.5). The combination of these three cross‐correlations is due to the triplet of SiOH groups in mutual spatial proximity (Figure 1 c). Some minor auto‐correlation intensity at SQ chemical shifts of approximately 1–3 ppm are assigned to randomly paired silanols at internal or external surface sites, and an autocorrelation at 4.3 ppm is due to physisorbed water and/or hydrogen‐bonded silanol pairs at other positions. In contrast, ITQ‐1 only shows three auto‐correlation signals with SQ chemical shifts of 1.0, 1.3, and 2.1 ppm, also assigned to external and internal surface silanols.

The cluster of three silanols in SSZ‐70 can be more directly unveiled in a 2D ^1^H triple‐quantum (TQ)‐SQ correlation experiment, Figure 2 c. This spectrum clearly highlights the SiOH triad: (3.1+4.2+5.5) ppm=12.8 ppm. The DQ autocorrelation signals at 1 to 3 and 4.2 ppm are absent in Figure 2 c, proving that they are due to pairs rather than triplets. In contrast, such a TQ‐SQ correlation experiment taken on ITQ‐1 yields no signal (not shown). Therefore, the silanol triads found in SSZ‐70 are absent in ITQ‐1.

The experimental finding that the three silanol groups in the nest yield distinct chemical shifts is further addressed via electronic‐structure calculations. We considered two structural motifs in DFT cluster calculations: a) a cyclic and b) an open triad with one OH‐bond bridging another Si‐O‐Si oxygen atom across an adjacent 5‐ring. The optimized structures are reported in Figure 3. The cyclic triad, Figure 3 a, retains a distorted, cyclic silanol structure, with hydrogen‐bond lengths of 1.70–1.80 Å (O⋅⋅⋅H) and O⋅⋅⋅O distances of 2.62–2.68 Å. The acyclic structure, Figure 3 b, is distorted to a greater extent. The hydrogen bond formed between the external SiOH group (O2b′′) and an Si‐O‐Si bridge (O7′′) is significantly longer (*d*(H⋅⋅⋅O)=2.35 Å) than the internal ones (*d*(H⋅⋅⋅O)=1.63, 1.69 Å), the internal ones on the other hand are shorter than in the cyclic structure. The calculated chemical shifts of the acyclic model of Figure 3 b are 3.10, 7.46, and 8.60 ppm. The span of these chemical shifts considerably exceeds the experimental ones. In terms of energy, this open silanol triad is 5.8 kcal mol^−1^ less stable than the cyclic form. Notably, the chemical shifts of the cyclic cluster model are not equivalent (6.35, 7.17, 7.68 ppm), and their span agrees much better with the experimental values. Therefore, we propose that the triad nests in SSZ‐70 are best described by a cyclic model, although the DFT calculations overestimate the strengths of the hydrogen bonds. However, some contribution of the acyclic model possibly in equilibrium with the cyclic structure appears to be feasible.

**Figure 3:**
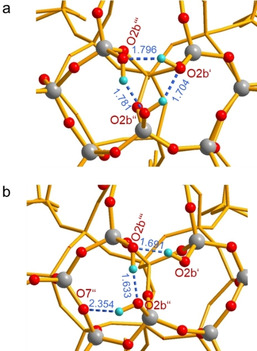
DFT‐optimized structures (PBE‐D3/def2‐SVP) of two cluster models of the silanol triad in SSZ‐70, a) cyclic, b) open triad; cyan H, red O, gray Si. Bond lengths in [Å]. Full cluster models with all 65 T atoms are shown in the Supporting Information.

The inequivalence of the three Q^3^ groups of the triad was previously observed by ^29^Si NMR spectroscopy.^12^ To explain this distortion of the crystallographic trigonal symmetry at the defect sites, the atomic occupancies of silanol monads and triads (Figure 1 c) are considered. A mixture of the two models breaks the trigonal symmetry in the layer (Figure S5), and this effect is suggested to be the reason that the three silanols in the nests become inequivalent. The resolution of the coupling pattern in Figure 2 a,c suggests that the monad/triad distribution follows some order within the layer. Interestingly, Berkson et al. recently reported some ordering of Al in Al‐SSZ‐70.^16^ This suggests that such ordering is a result of charge ordering occurring during the synthesis under the influence of the structure directing agent (SDA).

The rationale for the formation of the SiOH triad in SSZ‐70 is that the all‐silica material needs to accommodate negative charge in order to balance the *N*,*N*′‐diisobutyl imidazolium cation that is used as SDA in the synthesis of the material.^17^ The defect sites in as‐synthesized all‐silica zeolites are well known to consist of SiOH⋅⋅⋅⋅^−^OSi hydrogen bonds.^10^, ^18^ Either two or three SiOH donors interact with a charged siloxy (^−^OSi) acceptor, and the ^1^H chemical shifts in such hydrogen bonds are typically at around 10 ppm (Figure S6). The DQ‐SQ data of as‐synthesized Si‐SSZ‐70 (Figure S6) clearly reveal an autocorrelation signal (10.3+10.3=20.6 ppm), but a TQ‐SQ autocorrelation signal is absent (near 10+10+10=30 ppm, data not shown). It thus follows that there are two silanols in a nest for the as‐synthesized material. The interaction of the SDA with the defect sites can be clearly identified here by ^1^H DQ‐SQ MAS NMR (Figure S6).

This 2 SiOH+SiO^−^ cluster is converted into the SiOH triad in the calcined material (see above), where the SDA cation is absent and a negative charge is no longer needed. The different chemical shifts of silanol groups in the as‐synthesized and calcined materials are due to the different hydrogen bond acceptor properties, that is a siloxy group, SiO^−^, in the as‐synthesized and a silanol group (or Si‐O‐Si moiety) in the calcined materials. It should be noted that the stability of a SiOH triad (Figure 1 c), is not expected, and silanol condensation may, in general, take place upon calcination.5b, 6e However, the SiOH triad is located in a special position in SSZ‐70, where condensation of any two of the silanols would inevitably form a 3‐ring. Such 3‐rings are unknown in all‐silica zeolites, which is obviously due to the high ring strain.

These results on SSZ‐70 are in clear contrast to ITQ‐1, which does not have SiOH triads upon calcination. The defects with negative framework charge in as‐synthesized ITQ‐1, indicated by a ^1^H line at 10 ppm for the hydrogen bonds (Figure S6 b), are instead converted into a distinct defect site structure, which shows only pairs of silanols at chemical shifts lower than for SSZ‐70. This is striking, as the defect Q^3^ sites in as‐made all‐silica zeolites often occur in a ratio of 4:1 with respect to the charge of the SDA (three SiOH hydrogen‐bonded to one ^−^O‐Si yields four Q^3^ sites per charge).^17^ This observation is explained by a condensation of a considerable portion of the defect silanols in ITQ‐1 upon calcination, and then the material has silanols with no hydrogen bonding in a nest.

The variety of defect sites was further explored by extracting boron from B‐SSZ‐70. It is evident that sharp ^29^Si MAS NMR signals are found in the all‐silica material (Figure S1 a), reflecting the highly ordered silanol location. In contrast, deboronated B‐SSZ‐70 shows much broader resonances, Figure S1b. This indicates that boron is located at different positions in the framework (see ^11^B MAS NMR in Figure S2 a), and results in a disordered location of defect sites upon boron removal. ^1^H DQ‐SQ data of B‐SSZ‐70 (Figure S7 a) and of the deboronated material, deB‐B‐SSZ‐70 (Figure S7 b) reveal that the situation is in clear contrast to all‐silica SSZ‐70. The silanol triad, as found for all‐silica SSZ‐70, is absent in deB‐B‐SSZ‐70. This finding is easily explained by the negative charge of BO_4/2_^−^ in B‐SSZ‐70, making silanol nests to accommodate negative charge redundant. The deboronated material reveals silanol pairs (dyads) by a cross‐correlation signal in the ^1^H DQ‐SQ correlation experiment (Figure S4 b). Notably, more than two silanols cannot be confirmed (no triads or tetrads), because a TQ‐SQ correlation signal is absent (Figure S7 c). We conclude that removal of boron by acid leaching must lead to silanol condensation and transformation of the SiOH tetrads to a defect cluster with only two silanols. These results confirm the doubts that were raised by Senderov et al. as to the stability of a nest with four silanol groups.6e

We further measured FTIR spectra corresponding to Si‐SSZ‐70, deboronated B‐SSZ‐70, ITQ‐1, and Aerosil 200 (amorphous silica), after pretreating each material at 250 °C and subsequently cooling down to room temperature under vacuum. The data in the Figure S1 show the O‐H stretching vibration region of the infrared spectrum. On one extreme, we observe dehydroxylated Aerosil 200 (dehydroxylated at 500 °C) to exhibit a narrow band at 3740 cm^−1^, which is known to be representative of isolated terminal silanols. Silanols that are hydrogen bonded will generally tend to exhibit a lower frequency O‐H stretching vibration,^19^ and all crystalline materials contain such hydrogen‐bonded silanols. The material with the highest intensity of low energy bands in this region is Si‐SSZ‐70. This is consistent with Si‐SSZ‐70 being the only material with a silanol triad. To assess differences in acid strength, we adsorbed pyridine and followed its desorption under vacuum at various temperatures via FTIR spectroscopy on calcined SSZ‐70, deB‐B‐SSZ‐70, ITQ‐1, and amorphous silica. In all materials, the same two infrared bands (1596 cm^−1^ and 1445 cm^−1^) were observed for hydrogen‐bonded pyridine (no pyridinium cation).^20^ Compared with amorphous silica, deB‐B‐SSZ‐70, and ITQ‐1, which all released 90 % or more of adsorbed pyridine at 373 K, SSZ‐70 was unique in terms of its ability to retain a significant fraction—more than 30 %—of adsorbed pyridine at the same temperature. This higher affinity of pyridine to SSZ‐70 is consistent with the expected greater acidity of such a silanol triad nest, and is comparable to that of hydrogen‐bonded OH networks in molecules.^21^

In conclusion we have confirmed the existence of a silanol defect site nest with 3 SiOH groups in calcined zeolite SSZ‐70. This defect site is negatively charged in the as‐made material to balance the organic structure‐directing agent. Silanol condensation is suggested to be inhibited by the high strain that would take place in the resulting 3‐rings. In contrast silanol condensation is not inhibited upon calcination of all‐silica ITQ‐1 or boron removal from B‐SSZ‐70. These findings highlight that the stability and cluster size of silanol nests depends on their local framework environment, and silanol triads or tetrads are not expected to be stable in general for other zeolites.

## Conflict of interest

The authors declare no conflict of interest.

## Supporting information

As a service to our authors and readers, this journal provides supporting information supplied by the authors. Such materials are peer reviewed and may be re‐organized for online delivery, but are not copy‐edited or typeset. Technical support issues arising from supporting information (other than missing files) should be addressed to the authors.

Supplementary
