# Supplementary material for: A Stable Silanol Triad in the Zeolite Catalyst SSZ‐70
Source: Angew Chem Int Ed Engl. 2020 Apr 23;59(27):10939–43. doi: 10.1002/anie.202001364 (PMC7317713; doi:10.1002/anie.202001364)
Supplement: Supplementary file 1 — Supplementary [file ANIE-59-10939-s001.pdf]

Angewandte Chemie
Eine Zeitschrift der Gesellschaft Deutscher Chemiker

Supporting Information

A Stable Silanol Triad in The Zeolite Catalyst SSZ-70

Christian Schroeder, Christian Mück-Lichtenfeld, Le Xu, Nicolás A. Grosso-Giordano, Alexander Okrut, Cong-Yan Chen, Stacey I. Zones, Alexander Katz,* Michael Ryan Hansen, and Hubert Koller**

anie_202001364_sm_miscellaneous_information.pdf

Contents

A Experimental Section 2

A.1 Syntheses 2

A.2 Solid State NMR 2

A.3 DFT Calculations 2

B NMR data of calcined SSZ-70 3

B.1 ^{29}Si and ^{1}H MAS NMR 3

B.2 ^{11}B MAS NMR 4

B.3 ^{1}H DQ-SQ MAS NMR data of calcined all-silica SSZ-70 5

C Models 6

C.1 Results of DFT calculations 6

C.2 Schematic defect distribution models 7

D NMR data of as-synthesized SSZ-70, ITQ-1 and calcined B-SSZ-70 8

D.1 ^{1}H MAS NMR of as-made SSZ-70 and ITQ-1 and DQ-SQ MAS NMR of as-made SSZ-70 8

D.2 ^{1}H DQ-SQ and TQ-SQ MAS NMR spectra of B-SSZ-70 and deB B-SSZ-70 9

E FTIR spectroscopy 10

E.1 Experimental 10

E.2 Results 10

E.3 FTIR spectroscopy discussion 15

References 15

A Experimental Section

A.1 Syntheses

SSZ-70
Into a tared cup 2.08 grams of Tetraethyl orthosilicate (10 mMoles of SiO_2) is placed. Also 5 mMoles of N,N' diisobutyl imidazolium hydroxide is added in a 11.11 grams of reagent solution. The cup is kept closed for 3 days to let the TEOS hydrolyze and then the top is opened with the cup in a hood and ethanol and water are allowed to evaporate at room temperature. When the mass of solids in the cup is down to 2.82 grams, then concentrated (48%) HF is added; 5 mMoles are added in and the reaction is closed up and heated at 150 °C with 43 RPM rotation of the Parr reactor attached on a spit inside a Blue M convection oven. After 12 days there is a nice crystalline product which is then washed up with water at room temperature, dried and submitted for X-ray diffraction characterization. Calcination was carried out in air at 823 K for 6 h after a 1 K/min ramp.
B-SSZ-70
The sample preparation follows the recently published protocol.[1] The B-SSZ-70 product was calcined by treatment under air at 823 K for 6 h after a 1 K/min ramp. B removal was performed by treatment of 500 mg of the calcined product with 50 mL of 2N HNO_3 at 373 K for 18 h, followed by filtration and washing with copious amounts of H_2O.

ITQ-1
ITQ-1 was synthesized following a published procedure.[2] 5.5705 g of trimethyladamantammonium hydroxide (TMAdaOH) solution (0.56 mmol/g), 0.3853 g of hexamethyleneimine (HMI, from Aldrich), 0.1465 g of NaCl (from EM) and 5.0 g of H_2O are mixed under stirring to make a clear solution. Then 0.75 g of Aerosil 200 (from Degussa) is added and stirred at room temperature for 1 hour. The gel is then transferred to a Teflon-lined autoclave and heated at 150 °C under static conditions for 12 days. The resulting ITQ-1 material is then washed up with water at room temperature, dried and submitted for X-ray diffraction characterization.
To remove the occluded template molecules, the as-synthesized ITQ-1 powder sample was calcined in an air stream. The temperature stages were as follows: first from room temperature to 125 °C at 50 °C/h and hold for two hours, then to 540 °C at 50 °C/h and hold for 10 hours, subsequently to 595 °C at 50 °C/h with a final hold for 10 hours.

A.2 Solid State NMR

Prior to the NMR experiments, all samples were dried at 120°C under dynamic vacuum. NMR spectra were acquired on a Bruker Avance I spectrometer operating at a magnetic flux density of 9.4 T utilizing the software Topspin 1.3. The samples were packed into 4 mm ZrO_2 rotors with a Kel-F end cap under dry nitrogen atmosphere. ^{1}H MAS, ^{1}H DQ-SQ MAS and ^{1}H TQ-SQ MAS NMR spectra were carried at either 9 or 12.5 kHz MAS rate. Referencing was performed with adamantane ($\delta(^1H)$ = 1.78 ppm), and a $\pi/2$ pulse of 4 μs was used in all cases (effective rf field strength of 62.5 kHz). The recycle delay for ^{1}H MAS NMR was set to 20 s, and for multi-quantum experiments 4 s. Both for the ^{1}H-^{1}H DQ-SQ MAS and ^{1}H-^{1}H TQ-SQ MAS NMR spectra, the back-to-back pulse sequence was used as excitation and reconversion blocks, one rotor period each.
^{11}B MAS NMR spectra were referenced using liquid BF_3*Et_2O ($\delta(^{11}B)$ = 0 ppm), and a pulse length of 0.5 μs was used ($\pi/16$ pulse of the liquid standard, rf field strength 62.5 kHz). The recycle delay was set to 4 s. The sample was spun at a MAS rate of 12.5 kHz.
^{29}Si MAS NMR spectrum of deboronated SSZ-70 was referenced with tetrakis-trimethysilylsilane ($\delta(^{29}Si)$ = -9.8 ppm) with a $\pi/2$ pulse of 4.8 μs (52 kHz rf field strength). A recycle delay of 60 s was used. The MAS rate was 12.5 kHz.
^{29}Si MAS NMR spectrum of Si-SSZ-70 was acquired on a Bruker DSX500 spectrometer console operating with a 4.7 T magnet. The sample was filled in a 7 mm ZrO_2 rotor with Kel-F end cap and was spun with 4 kHz. Referencing was performed with tetrakistrimethysilylsilane, and a $\pi/2$ pulse of 8 μs was utilized. A recycle delay of 60 s was used.

A.3 DFT Calculations

We performed DFT calculations elucidating alternative local structures of silanol triplets, using the GGA functional PBE[3] together with Grimmes D3 dispersion correction [4] and a double zeta basis set (def2-SVP).[5] We constructed a cluster model around the silanol center from the crystal structure by replacing Si-O bonds on the edge of the cluster with Si-H and keeping the positions of these terminal hydrogen atoms fixed in the optimization (for details see SI, Fig. S4). All other atomic positions were fully optimized. Two structures, one with a cyclic silanol triplet and one with an open silanol triplet (one hydrogen pointing towards an Si-O-Si oxygen atom) were chosen as models for comparison.
The relative (electronic) energies were recalculated with a triple zeta basis set (def2-TZVP)[5] and a hybrid meta GGA functional (PW6B95)[6] to eliminate errors in the hydrogen bond energy due to the small basis set used for optimization. All DFT calculations were performed with Turbomole 7.3.[7]

B NMR data of calcined SSZ-70

B.1 ^{29}Si and ^{1}H MAS NMR

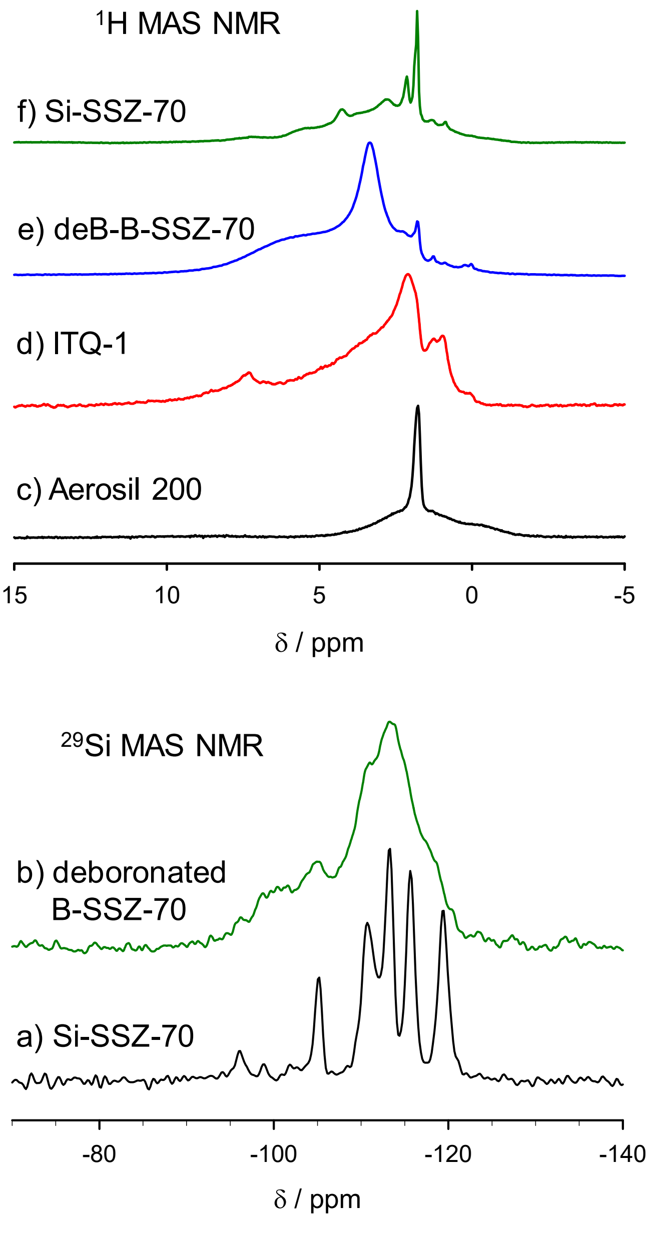

Figure S1: a,b) ^{29}Si MAS NMR and c-f) ^{1}H MAS NMR spectra of calcined samples, which were dehydrated at 120°C; a) calcined all-silica SSZ-70 and b) deB-B-SSZ-70; chemical shifts between -95 and -103 ppm are assigned to Q^3 groups and from -103 to -120 ppm to Q^4 framework sites.[8] Deboronated SSZ-70 shows much broader lines, because boron is distributed with reduced occupancies among several crystallographic sites. Therefore, its removal generates disorder and thus line broadening. In contrast, the Q^3 sites in Si-SSZ-70 are ordered at well-defined crystallographic sites.

B.2 ^{11}B MAS NMR

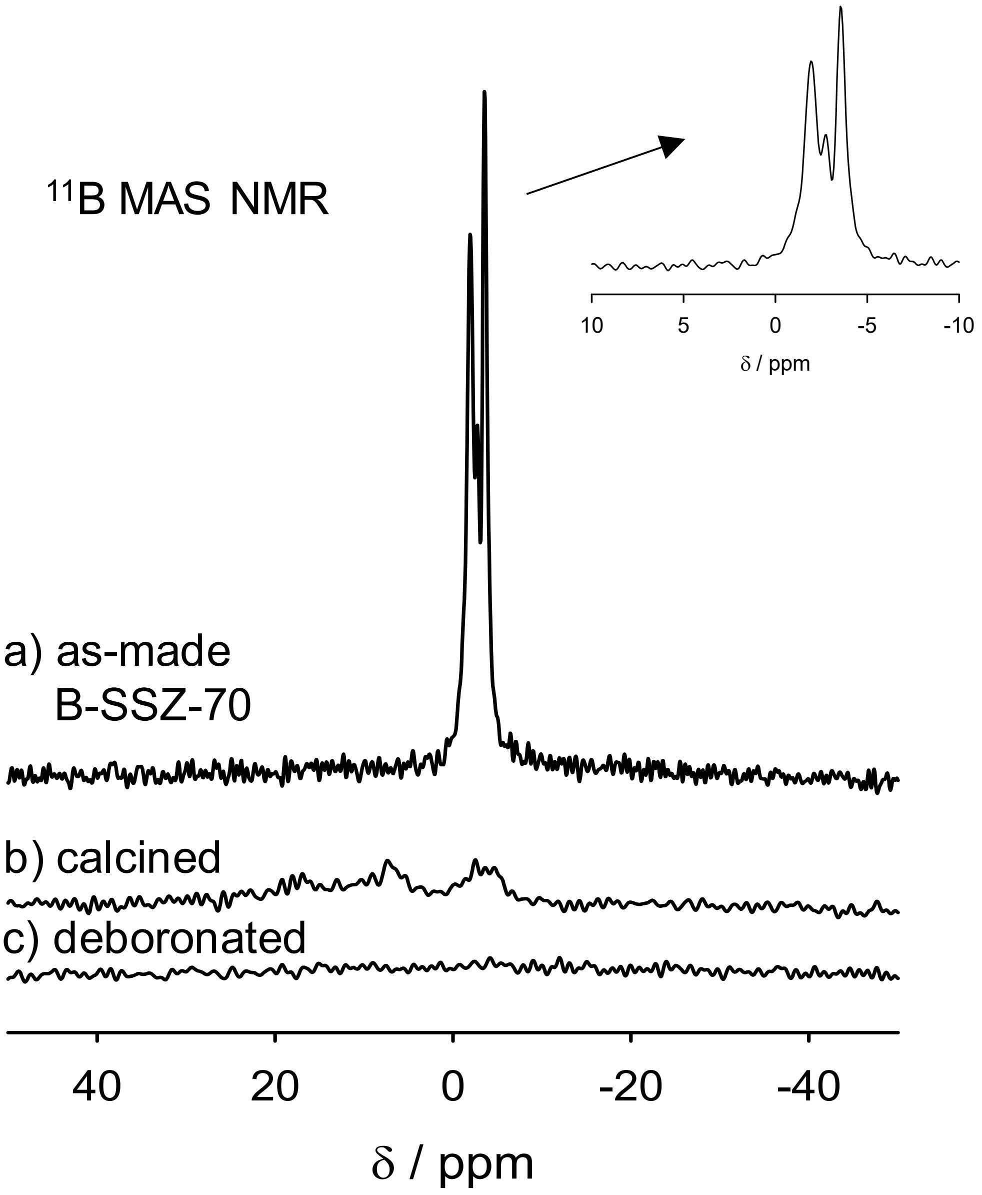

Figure S2: ^{11}B MAS NMR spectra of a) as-made B-SSZ-70, b) calcined B-SSZ-70 and c) deB-B-SSZ-70. Boron occupies at least three distinct sites in the as-made zeolite framework, see inset in part a). The calcined sample shows a very broad signal, which is due to trigonal boron in the framework, and unresolved with respect to different sites. Such trigonal boron species can be easily extracted by acid leaching, which is demonstrated in part c).

B.3 ^{1}H DQ-SQ MAS NMR data of calcined all-silica SSZ-70

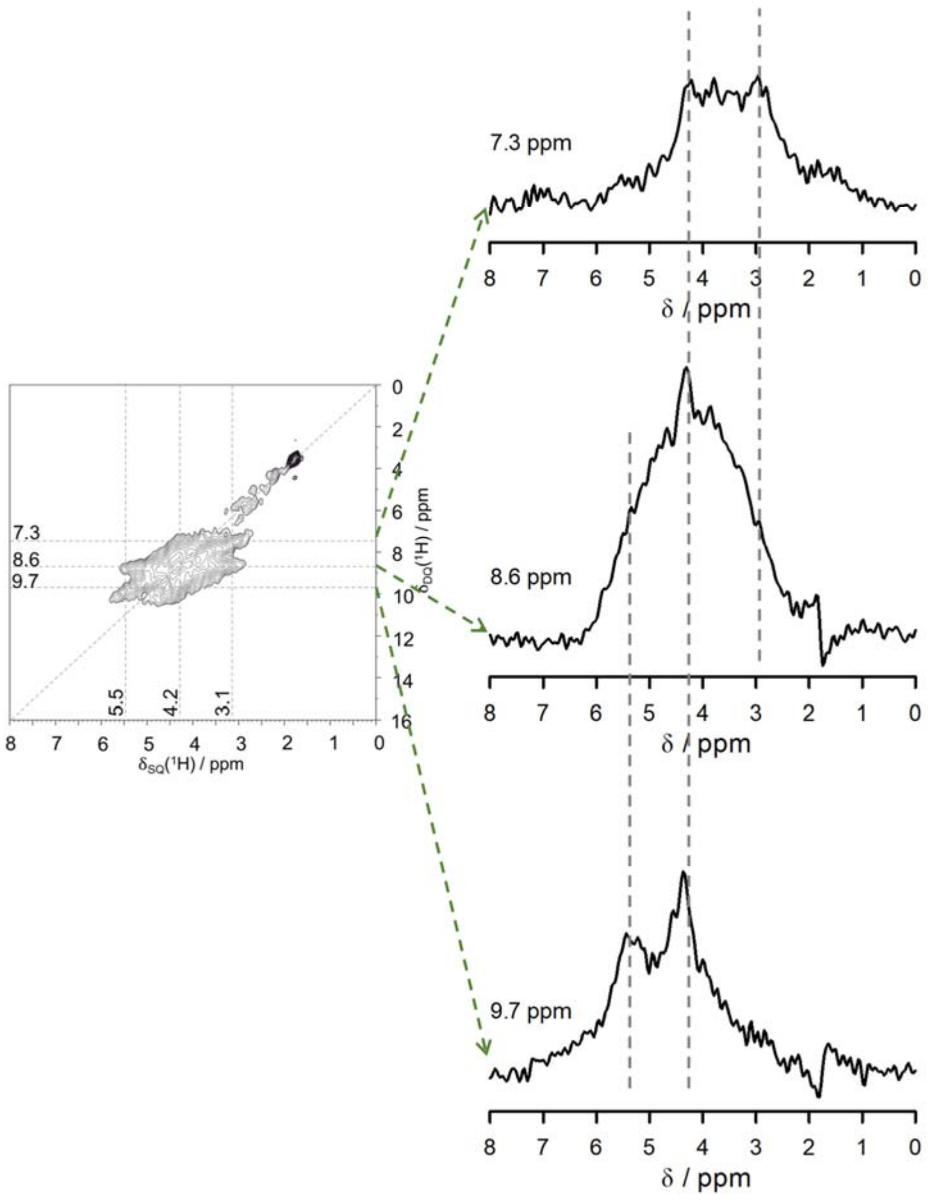

Figure S3: 1D slices taken at 7.3, 8.6 and 9.7 ppm of the ^{1}H DQ-SQ MAS NMR spectrum of calcined all-silica SSZ-70. Two cross-correlations at 7.3 ppm (3.1+4.2) and 9.7 ppm (4.2+5.5) are indicated by dashed vertical lines. The expected cross-correlation at 8.6 ppm (3.1+5.5) in the silanol triad is superimposed with an auto-correlation (4.3+4.3), which is likely due to residual water molecules and/or paired H-bound silanols at other positions.

C Models

C.1 Results of DFT calculations

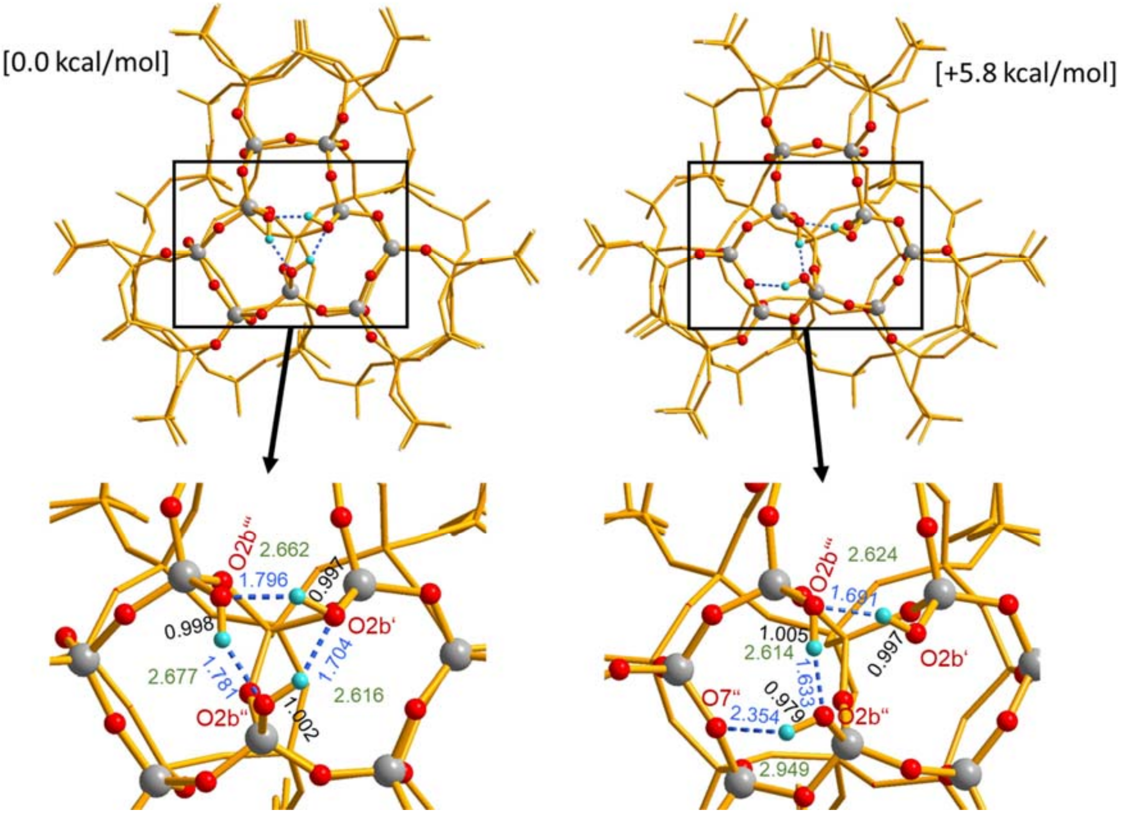

Figure S4: Optimized cluster models (PBE-D3/def2-SVP) of the closed (symmetric, left) and open (asymmetric) model (right) of the triple silanol nest of calcined all-silica SSZ-70. The asymmetric model is less stable by 5.8 kcal/mol (PW6B95-D3/def2-TZVP//PBE-D3), and the cyclic model in a) is favorable (see also main text for further discussion). The defect site oxygen atoms are created from the crystallographic O2b positions and become inequivalent after geometry optimization. H····O distances (blue), H-O distances (black) and O····O distances (green) are indicated in Å.

Relative energies of the two structures

Method	ΔE = E(closed $(SiOH)_3$) – E(cyclic) $(SiOH)_3$) / kcal/mol
PBE-D3/def2-SVP (M1)	+9.1
PBE-D3/def2-TZVP // M1	+7.1
PW6B95-D3/def2-TZVP // M1	+5.8

C.2 Schematic defect distribution models

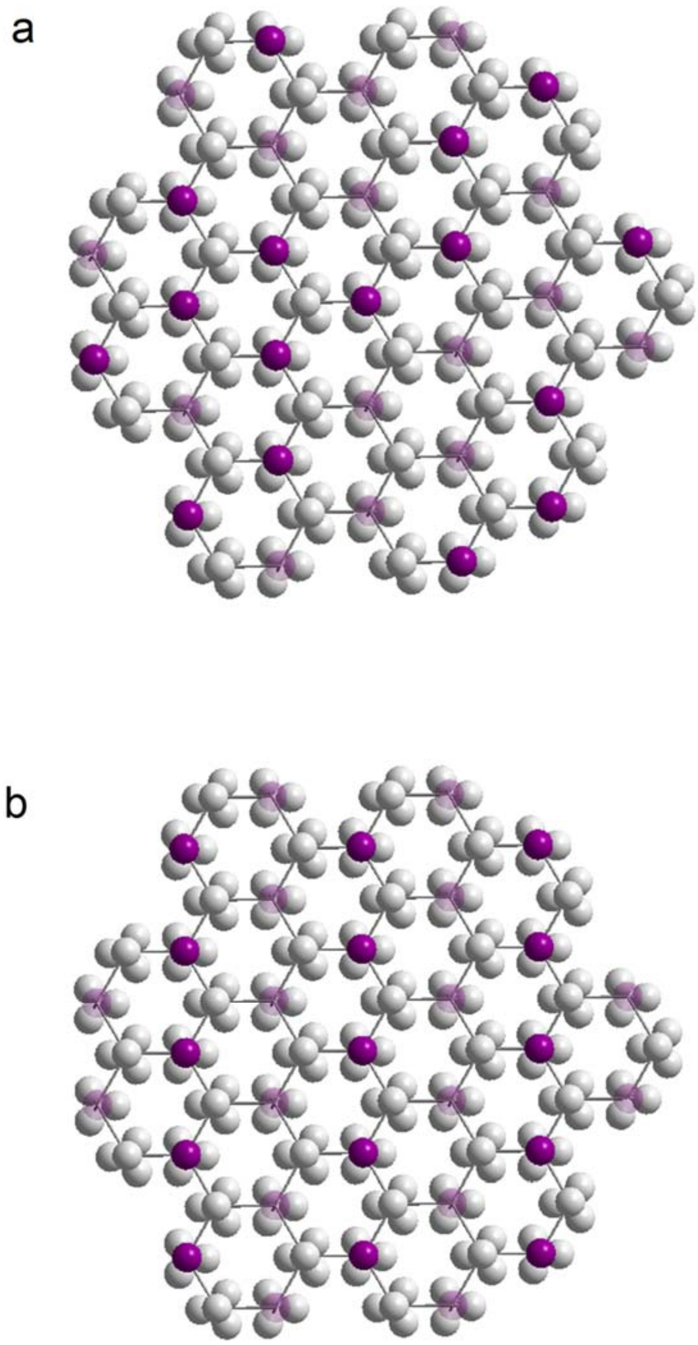

Figure S5: a) disordered and b) ordered distribution models of SiOH triads. The hexagonal pattern represents the topological arrangement of MWW cages of a layer in zeolite SSZ-70. Grey balls on the surface indicate MWW cages that connect to the next layer, and colored balls are unconnected sites (silanol monads or triads). The bold violet balls represent occupied dangling SiOH groups (monads), and at the pale violet balls, these SiOH groups are vacant, to yield a SiOH triad.

D NMR data of as-synthesized SSZ-70, ITQ-1 and calcined B-SSZ-70

D.1 ^{1}H MAS NMR of as-made SSZ-70 and ITQ-1 and DQ-SQ MAS NMR of as-made SSZ-70

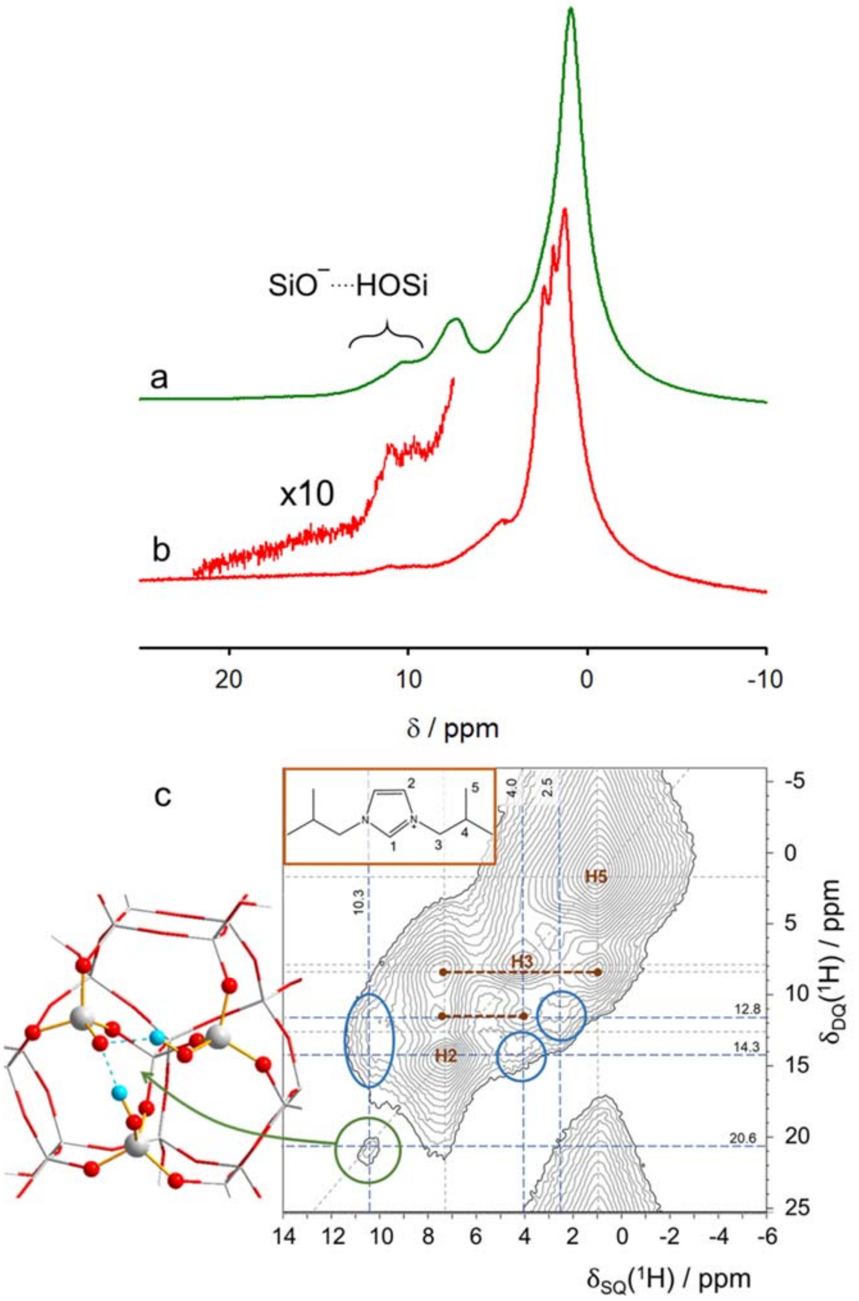

Figure S6: ^{1}H MAS NMR spectra of a) as-made SSZ-70 and b) as-made ITQ-1; c) ^{1}H SQ-DQ MAS NMR spectrum of as-made all-silica SSZ-70. The model illustrates the two silanol groups interacting with a charged SiO^- moiety via hydrogen bonds (observed at 10.3 ppm) to balance the cationic charge of the organic structure directing agent, SDA (N,N'-diisobutyl imidazolium). Cross-correlations between the SDA and the silanol groups of the charged defect site are indicated in blue framing. Orange colored labels indicate the auto-correlations of respective hydrogen atoms in the SDA. H1 and H4 do not appear as auto-correlations, because these are single atoms. Horizontal orange lines indicate cross-correlations between H2 and H3 as well as H2 and H5.

D.2 ^{1}H DQ-SQ and TQ-SQ MAS NMR spectra of B-SSZ-70 and deB B-SSZ-70

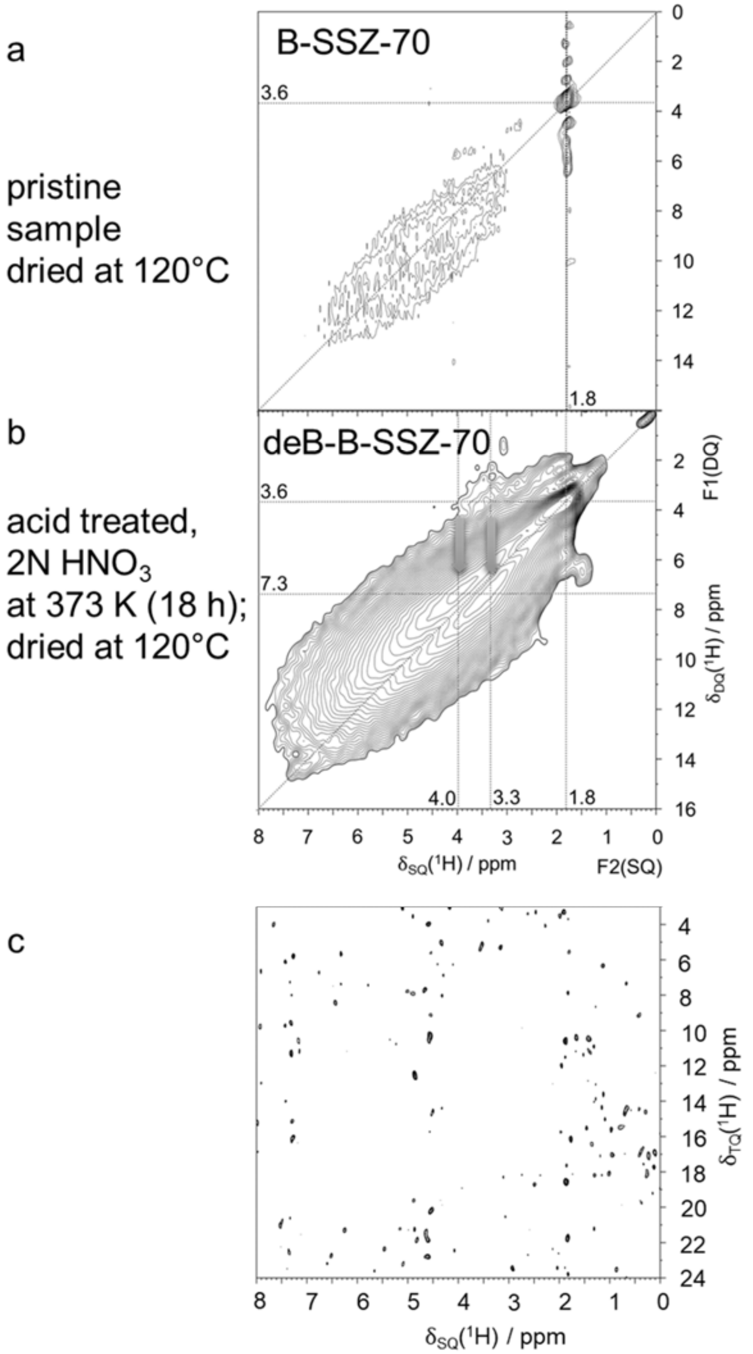

Figure S7: a) ^{1}H DQ-SQ ^{1}H MAS NMR of B-SSZ-70, and b) of the deboronated sample, c) TQ-SQ ^{1}H MAS NMR of the deboronated sample. Spectrum a) shows an autocorrelation for paired external surface silanol groups at δ_{SQ} = 1.8 ppm and a broad signal for a small portion of hydrogen-bonded, paired silanols at 3-7 ppm. Spectrum b) shows a cross-correlation for paired H-bonded silanols (arrows) along with an autocorrelation for external surface silanols at 1.8 ppm. Spectrum c) shows that the pairs in b) have no other neighbor, so these silanols are no more than two. Spectra in a) and b) were previously published,[1] and shown for comparison with c).

E FTIR spectroscopy

E.1 Experimental

IR spectra were collected on a Nicolet 6700 FTIR spectrometer. The IR spectra of adsorbed pyridine were recorded as follows: a self-supported wafer was set in a quartz IR cell sealed with CaF_2 windows, where it was evacuated at 523 K for 1.5 h (i.e. sufficient time to dehydrate sample) before pyridine adsorption. Pyridine adsorption was conducted by exposing the wafer to pyridine vapor at its saturation vapor pressure at 303 K. This was accomplished by injecting an excess amount of pyridine under N_2 atmosphere via septum into a side port of the in-situ cell, and allowing the vapor phase of this injected pyridine to equilibrate with the wafer for 1 h (i.e. no change in the FTIR spectrum was observed after 1 min following pyridine injection). Physisorbed pyridine was removed by evacuation at 323 K, 373 K, and 423 K for 0.5 h (minimal rate of change in the FTIR spectrum was observed after 30 min), and data collection followed at each temperature.

E.2 Results

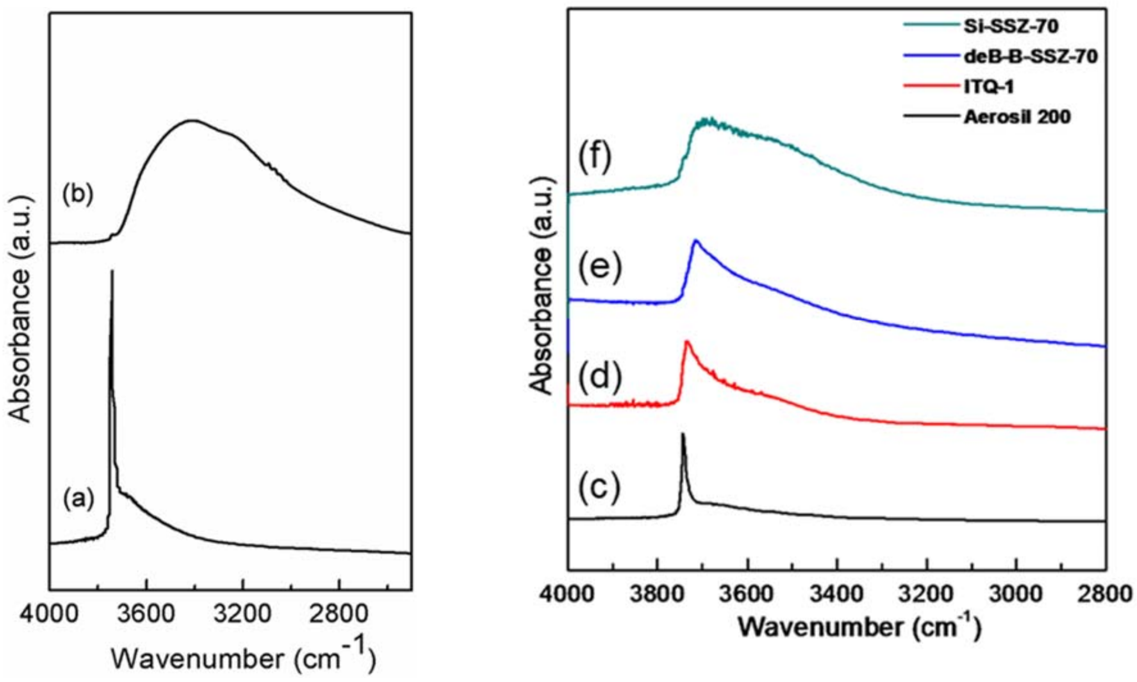

Figure S8: In-situ FTIR spectra of dehydroxylated amorphous silica (Aerosil-200 calcined at 773 K in air) during the following treatments: (a) evacuation at 523 K and (b) pyridine adsorption at 303 K after (a). FTIR spectra of (c) Aerosil 200 (amorphous silica), (d) ITQ-1, (e) deboronated B-SSZ-70 and (f) Si-SSZ-70, after pretreating each material at 250 °C and subsequently cooling down to room temperature under vacuum.

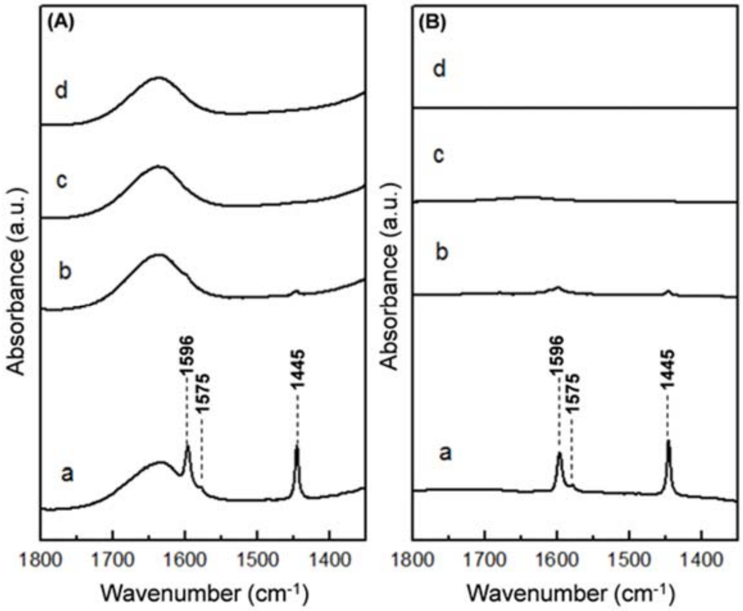

Figure S9: In-situ FTIR spectra of pyridine adsorbed on dehydroxylated amorphous silica (Aerosil-200 calcined at 773 K). (A) raw data corresponding to (a) pyridine adsorption at 303 K followed by its desorption at (b) 323 K, (c) 373 K and (d) 423 K. (B) FTIR difference spectra calculated by subtracting the spectrum in (d) above from corresponding data in (a), (b), (c), and (d) above (the FTIR difference spectrum in (B) shares the same label as the raw data at the same temperature in (A)).

Table S1: Normalized infrared band peak areas for dehydroxylated amorphous silica (Aerosil-200 calcined at 773 K in air) based on the FTIR difference spectra in Figure S9B.

FTIR Difference	Relative peak area (%)		
Spectrum	1596 cm^{-1}	1575 cm^{-1}	1445 cm^{-1}
Figure S9Ba	100	100	100
Figure S9Bb	11.7 ± 1.2	0	9.4 ± 0.6
Figure S9Bc	0	0	0
Figure S9Bd	0	0	0

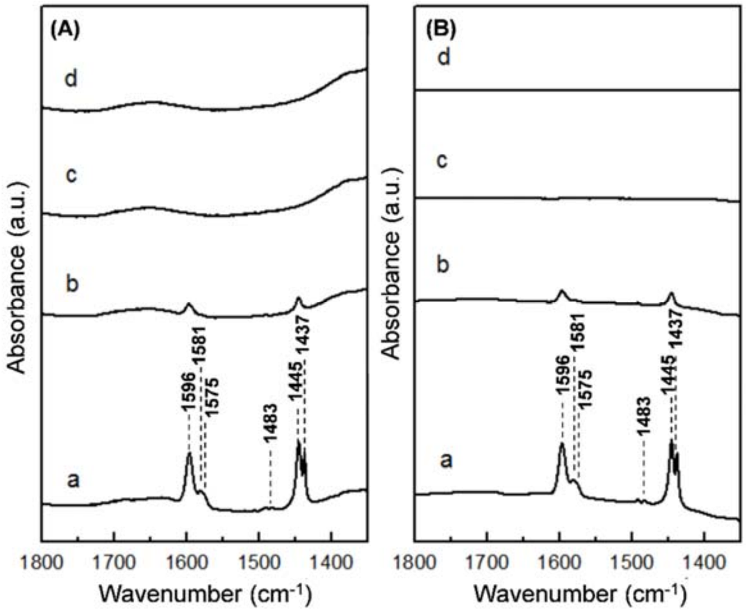

Figure S10: In-situ FTIR spectra of pyridine adsorbed on deB-B-SSZ-70. (A) raw data corresponding to (a) pyridine adsorption at 303 K followed by its desorption at (b) 323 K, (c) 373 K and (d) 423 K. (B) FTIR difference spectra calculated by subtracting the spectrum in (d) above from corresponding data in (a), (b), (c), and (d) above (the FTIR difference spectrum in (B) shares the same label as the raw data at the same temperature in (A)).

Table S2: Normalized infrared band peak areas for deB-B-SSZ-70 based on the FTIR difference spectra in Figure S10B.

FTIR Difference	Relative peak area (%)					
Spectrum	1596 cm^{-1}	1581 cm^{-1}	1575 cm^{-1}	1483 cm^{-1}	1445 cm^{-1}	1437 cm^{-1}
Figure S10Ba	100	100	100	100	100	100
Figure S10Bb	22.3 ± 0.8	0	0	0	25.4 ± 0.7	0
Figure S10Bc	0	0	0	0	0	0
Figure S10Bd	0	0	0	0	0	0

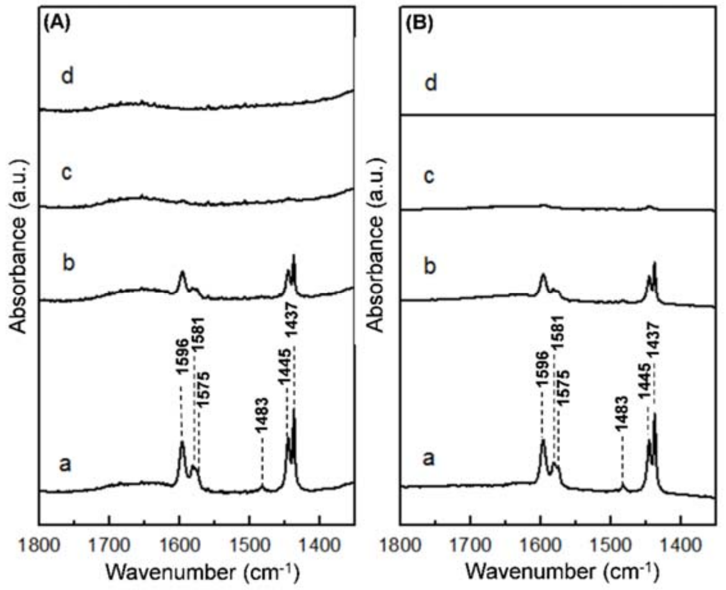

Figure S11: In-situ FTIR spectra of pyridine adsorbed on ITQ-1. (A) raw data corresponding to (a) pyridine adsorption at 303 K followed by its desorption at (b) 323 K, (c) 373 K and (d) 423 K. (B) FTIR difference spectra calculated by subtracting the spectrum in (d) above from corresponding data in (a), (b), (c), and (d) above (the FTIR difference spectrum in (B) shares the same label as the raw data at the same temperature in (A)).

Table S3: Normalized infrared band peak areas for ITQ-1 based on the FTIR difference spectra in Figure S11B.

FTIR Difference	Relative peak area (%)					
Spectrum	1596 cm^{-1}	1581 cm^{-1}	1575 cm^{-1}	1483 cm^{-1}	1445 cm^{-1}	1437 cm^{-1}
Figure S11Ba	100	100	100	100	100	100
Figure S11Bb	63.5 ± 0.4	37.5 ± 0.2	30.1 ± 0.7	25.4 ± 0.3	54.0 ± 0.4	47.8 ± 1.1
Figure S11Bc	9.1 ± 0.6	0	0	0	8.0 ± 0.5	0
Figure S11Bd	0	0	0	0	0	0

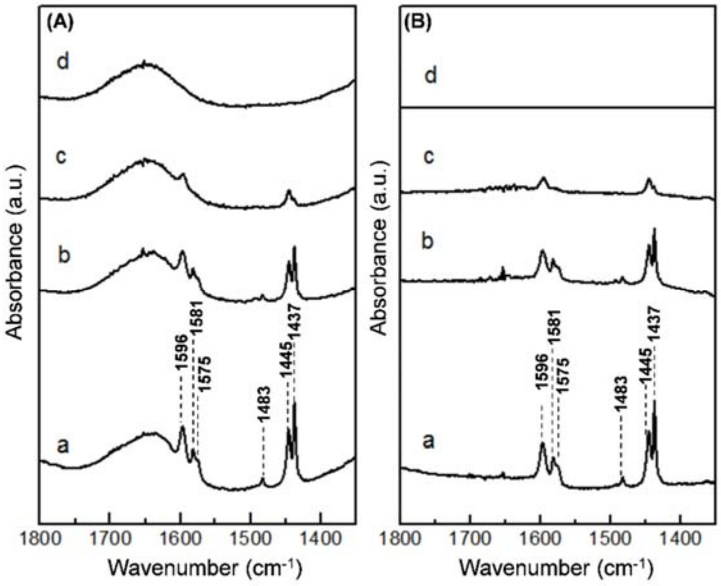

Figure S12: In-situ FTIR spectra of pyridine adsorbed on SSZ-70. (A) raw data corresponding to (a) pyridine adsorption at 303 K followed by its desorption at (b) 323 K, (c) 373 K and (d) 423 K. (B) FTIR difference spectra calculated by subtracting the spectrum in (d) above from corresponding data in (a), (b), (c), and (d) above (the FTIR difference spectrum in (B) shares the same label as the raw data at the same temperature in (A)).

Table S4: Normalized infrared band peak areas for SSZ-70 based on the FTIR difference spectra in Figure S12B.

FTIR Difference	Relative peak area (%)					
Spectrum	1596 cm^{-1}	1581 cm^{-1}	1575 cm^{-1}	1483 cm^{-1}	1445 cm^{-1}	1437 cm^{-1}
Figure S12Ba	100	100	100	100	100	100
Figure S12Bb	91.2 ± 0.4	77.2 ± 1.1	54.2 ± 0.8	46.2 ± 0.3	78.8 ± 0.6	81.0 ± 0.3
Figure S12Bc	32.0 ± 0.6	8.5 ± 0.3	0	0	37.6 ± 0.5	5.8 ± 0.7
Figure S12Bd	0	0	0	0	0	0

E.3 FTIR spectroscopy discussion

Dehydroxylated amorphous silica (Aerosil-200 calcined at 773 K in air) was used as a basis for a simple silica surface, because we have previously shown that such a silica comprises only silanols without hydrogen-bonds[1] and because the open nature of the silica surface minimizes adsorption effects in micropores. The FTIR spectrum of this silica sample is shown in Figure S8a, which exhibits a single prominent band in the hydroxyl region at 3740 cm^{-1}. Pyridine adsorption at 303 K causes complete disappearance of this band, and the appearance of a broad band in the range 3000 cm^{-1} – 3600 cm^{-1}. In the spectral window representative of pyridine, strong bands at 1596 cm^{-1} and 1445 cm^{-1}, and a weak band at 1575 cm^{-1} are observed, which is consistent with prior data for hydrogen-bound pyridine on silica.[9] However, as shown by data in Figure S9, at a desorption temperature of 323 K under vacuum, less than 15% of the adsorbed pyridine remains, with virtually no trace of adsorbed pyridine at a desorption temperature of 373 K.

For comparison, we also performed the same pyridine adsorption/desorption experiments for deB-B-SSZ-70, ITQ-1, and SSZ-70. The same behavior was observed for deB-B-SSZ-70 as described above for dehydroxylated amorphous silica, in terms of the same two infrared bands corresponding to hydrogen-bound pyridine, as shown in Figure S10. Slightly more pyridine retention was observed during desorption at 323 K for deB-B-SSZ-70 (Table S2) compared with amorphous silica in Table S1, and this can be rationalized on the basis of the expected paired hydrogen-bound silanols in deB-B-SSZ-70, as characterized previously by ^{1}H DQ-SQ MAS NMR spectrosocpy.[1] For both amorphous silica and deB-B-SSZ-70, we do not observe any pyridine remaining after a desorption temperature of 373 K, consistent with previous reports of hydrogen-bound pyridine on oxide supports.[10] In contrast, ITQ-1 retains a small fraction – less than 10% in Table S3 – of pyridine at a desorption temperature of 373 K, and exhibits the same two bands for adsorbed pyridine remaining at this temperature as observed for amorphous silica and deB-B-SSZ-70 at 323 K. However, SSZ-70 exhibits the highest retention of adsorbed pyridine, with over 30% remaining at 373 K in Table S4. This pyridine is also represented by the same two infrared bands as observed previously for the other materials above. Altogether, based on our data, we conclude that while the frequency of infrared bands of pyridine are not a sensitive reporter of the strength of pyridine adsorption, being the same for samples comprising isolated silanols as they are for samples containing extended silanol hydrogen-bond networks above, they are a reasonable reporter for the amount of pyridine adsorbed at a given desorption temperature. The significantly higher pyridine retention during desorption at 373 K signifies a higher adsorption affinity of pyridine to SSZ-70. Given the similarity of the framework structures of deB-B-SSZ-70, ITQ-1, and SSZ-70, our data is consistent with a greater hydrogen-bond donating ability of silanols in SSZ-70 – consistent with the unique existence of silanol triads in this framework as shown by ^{1}H TQ-SQ MAS NMR spectroscopy.

References

[1] N. A. Grosso-Giordano, C. Schroeder, A. Okrut, A. Solovyoy, C. Schöttle, W. Chassé, N. Marinkoyic, H. Koller, S. I. Zones, A. Katz, *J. Am. Chem. Soc.* **2018**, *140*, 4956-4960.
[2] M. A. Camblor, A. Corma, M. J. Diaz-Cabanas, C. Baerlocher, *J. Phys. Chem. B* **1998**, *102*, 44-51.
[3] J. P. Perdew, K. Burke, M. Ernzerhof, *Phys. Rev. Lett.* **1996**, *77*, 3865-3868.
[4] S. Grimme, J. Antony, S. Ehrlich, H. Krieg, *J. Chem. Phys.* **2010**, *132*, 154104.
[5] F. Weigend, R. Ahlrichs, *Phys. Chem. Chem. Phys.* **2005**, *7*, 3297-3305.
[6] Y. Zhao, D. G. Truhlar, *J. Phys. Chem. A* **2005**, *109*, 5656-5667.
[7] TURBOMOLE V7.3 2018, a development of University of Karlsruhe and Forschungszentrum Karlsruhe GmbH, 1989-2007, TURBOMOLE GmbH. http://www.turbomole.com (last accessed December 2019).
[8] S. Smeets, Z. J. Berkson, D. Xie, S. I. Zones, W. Wan, X. D. Zou, M. F. Hsieh, B. F. Chmelka, L. B. McCusker, C. Baerlocher, *J. Am. Chem. Soc.* **2017**, *139*, 16803-16812.
[9] E. P. Parry, *J. Catal.* **1963**, *2*, 371-379.
[10] M. I. Zaki, M. A. Hasan, F. A. Al-Sagheer, L. Pasupulety, *Colloids Surf. A - Physicochem. Eng. Asp.* **2001**, *190*, 261-274.
